# Communication, privacy and interpersonal care in non-obstetric gynaecological examinations: a scoping review

**DOI:** 10.3389/fmed.2026.1992943

**Published:** 2026-10-21

**Authors:** Xiaoting Yan, Yue Zhang, Yunhuan Sun, Tingting Cao, Han Xu

**Affiliations:** Lianyungang Maternal and Child Health Hospital, Lianyungang, China

**Keywords:** communication, gynaecological examination, interpersonal care, patient experience, pelvic examination, privacy, scoping review

## Abstract

**Background:**

Non-obstetric gynaecological examinations are common but sensitive clinical encounters in which communication, privacy, bodily exposure, perceived control, and interpersonal conduct may shape women's experiences and engagement with care. Evidence across different examination contexts remains dispersed and unintegrated.

**Objective:**

To map communication, privacy, and interpersonal care processes reported in relation to women's experiences of non-obstetric gynaecological examinations, examine how these processes are distributed across stages of the encounter, and identify gaps in the evidence.

**Methods:**

Seven electronic databases were searched from inception to 4 September 2026, supplemented by the CNKI Dissertations Database and reference-list screening. Quantitative, qualitative, and mixed-methods primary studies were eligible. Two reviewers independently screened records and extracted data. Findings were synthesised narratively and mapped onto the pre-, intra- and post-examination phases of the encounter. The review was prospectively registered on the Open Science Framework and reported in accordance with PRISMA-ScR.

**Results:**

Twenty-eight studies from 11 countries were included, comprising 20 quantitative and eight qualitative studies. Pre-examination evidence focused on prior knowledge, procedural information, expectations, communication accessibility, and preparation. Intra-examination prominent care processes included privacy protection, management of bodily exposure, explanation, permission, perceived control, clinician conduct, clinician gender, and chaperone or companion preferences. Immediate responses included embarrassment, anxiety, fear, pain, discomfort, stress, and perceived vulnerability. Evidence concerning disclosure, willingness to return, follow-up, delayed attendance, and avoidance was comparatively sparse and heterogeneous. Across studies, preferences regarding clinician gender, chaperones, companions, and participation varied between women and settings.

**Conclusion:**

Women's experiences of gynaecological examinations extend beyond technical performance to the organisation and interpersonal conduct of the whole care encounter. Preparation, privacy protection, responsive communication, recognition of discomfort, and respect for individual preferences represent potentially modifiable components of gynaecological and interdisciplinary practice. Prospective and intervention research is needed to clarify how these processes influence subsequent care engagement.

## 1 Introduction

Non-obstetric gynaecological examinations are a routine component of women's health care and are undertaken for symptom assessment, cervical screening and follow-up, investigation of pelvic complaints, and other diagnostic purposes (1, 2). Many of these examinations involve visualisation, palpation, instrumentation, imaging, or exposure of the genital or pelvic area and therefore require women to negotiate intimate bodily exposure within a clinical encounter. Although clinically necessary, such examinations may evoke embarrassment, anxiety, fear, pain, discomfort, or a sense of vulnerability and loss of control (3–5). These concerns may be particularly salient for women with previous experiences of violence or trauma, for whom pelvic examination can present additional challenges to comfort and care engagement in gynaecological settings (6). Women's experiences of these examinations therefore encompass not only the technical conduct of the procedure but also how the encounter is explained, how bodily exposure and privacy are managed, and whether women feel respected, informed, and able to exercise some control over what happens (3, 7).

Communication, privacy, and interpersonal conduct are potentially modifiable components of the examination encounter. Information provided before an examination, opportunities to ask questions, explanation during the procedure, permission before intimate steps, adequate covering, and clinicians' responses to discomfort may shape how women experience care (3, 7). Conversely, limited explanation, insufficient privacy, rushed or insensitive behaviour, and lack of responsiveness may contribute to embarrassment, anxiety, discomfort, or difficulty expressing concerns. Broader research on clinician–patient communication suggests that communication can support trust, participation, understanding, and other care-related outcomes when patients are able to express preferences and participate in clinical interactions (7–9). Preferences regarding clinician gender, chaperones, companions, and the degree of participation or control may also vary between women and across clinical settings. These processes involve the wider clinical team: depending on the setting, physicians, nurses, midwives, sonographers, and other professionals may contribute to preparation, procedural communication, privacy protection, emotional support, and continuity of care (10–12). Examination-specific care processes are therefore relevant to broader principles of patient-centred gynaecological care, including respect for individual preferences, participation, responsiveness, and therapeutic communication (13–16).

Previous reviews have addressed related but narrower questions, including barriers to follow-up after abnormal cervical screening, barriers to cervical cancer screening, healthcare interactions surrounding abnormal menstruation, help-seeking for stigmatised pelvic health symptoms, and interventions intended to improve women's experiences of pelvic examinations (17–23). Of these, the recent systematic review by Mignot et al. (21) specifically evaluated the effectiveness of interventions designed to improve women's experiences of pelvic examinations. However, the broader distribution of communication, privacy, and interpersonal care processes across different non-obstetric gynaecological examination contexts has not been systematically mapped. It also remains unclear which potentially modifiable processes have received empirical attention at different stages of the encounter and where evidence remains limited, particularly in relation to subsequent care engagement.

Accordingly, this scoping review aimed to map communication, privacy, and interpersonal care processes reported in relation to women's experiences of non-obstetric gynaecological examinations and related intimate diagnostic procedures, examine how these processes and associated experiences are distributed across stages of the examination encounter, and identify patterns and gaps in the available evidence. The review focused on intimate examination-related encounters involving direct examination, visualisation, instrumentation, palpation, imaging, or exposure of the genital or pelvic area, rather than general gynaecological consultations or broader experiences of accessing women's health services. This scoping review makes three distinct, novel contributions to the published literature. First, it establishes a full-stage evidence mapping framework that organises findings across the pre-, intra-, and post-examination continuum. This framework integrates three interrelated core domains of communication, privacy, and interpersonal care, rather than focusing narrowly on a single outcome or intervention type. Second, it explicitly identifies evidence gaps relating to downstream care engagement, and challenges the widely held implicit assumption that negative examination experiences translate directly into subsequent avoidance of care. Third, it synthesises evidence drawn from 11 countries and multiple examination contexts, combining quantitative prevalence data with qualitative experiential insights to identify both cross-context commonalities and setting-specific variation.

## 2 Methods

### 2.1 Review design

This scoping review followed the methodological framework developed by Arksey and O'Malley (24), with refinements proposed by Levac et al. (25) and Daudt et al. (26). The framework guided the formulation of the review questions, identification of relevant studies, study selection, data charting, and the collation, synthesis, and mapping of the evidence. A scoping review was considered appropriate because the available literature encompassed varied examination types, clinical settings, study designs, care-process factors, and reported experiences or outcomes.

### 2.2 Protocol registration and reporting

The review is reported in accordance with the Preferred Reporting Items for Systematic Reviews and Meta-Analyses extension for Scoping Reviews (PRISMA-ScR) (27), with the completed checklist provided in Supplementary Material 1. The protocol was developed following established methodological guidance for scoping review protocols (28) and was prospectively registered on the Open Science Framework (OSF) on 7 April 2026 (DOI: 10.17605/OSF.IO/P8FQW); the core eligibility criteria and synthesis approach remained substantively unchanged throughout the review process. Two rounds of protocol amendment have been documented and timestamped in the OSF project record: On 11 April 2026, minor refinements to the title wording and elaboration of the three core review questions were implemented to improve conceptual clarity, with no alterations to eligibility criteria or synthesis methodology. On 4 September 2026, prior to manuscript submission, the search strategies were refined to enhance coverage of under-represented examination types and care-related concepts, and all final database searches were rerun from inception to this date. Specific refinements included: expansion of examination-specific search terms for colposcopy, transvaginal ultrasound, speculum examination and bimanual pelvic examination across all databases, extension of keywords relating to dignity, perceived control, continuity of care, chaperone support and interpersonal care to align with the review's three core conceptual domains, and optimisation of Chinese-language search strategies for CNKI and the CNKI Dissertations Database to improve sensitivity for grey literature. A supplementary search of the CNKI Dissertations Database was also conducted to expand grey-literature coverage, and the complete database-specific final search strategies are provided in full in Supplementary Material 2.

### 2.3 Search strategy

A comprehensive search was conducted across seven electronic databases: PubMed, Embase, CINAHL, Web of Science Core Collection, Scopus, PsycArticles via APA PsycNet, and China National Knowledge Infrastructure (CNKI). The final searches were conducted from database inception to 4 September 2026, with no restriction on publication year. Studies available in English or Chinese were eligible. Search strategies combined controlled vocabulary terms, where available, including MeSH and Emtree terms, with free-text keywords relating to gynaecological examinations, communication, privacy, interpersonal care, and relevant examination-related experiences and outcomes. The strategy was adapted for each database, and the complete database-specific search strategies are provided in Supplementary Material 2. In addition, the CNKI Dissertations Database was searched using an adapted Chinese-language strategy to identify potentially eligible grey literature. Records retrieved from the seven electronic databases and the CNKI Dissertations Database were imported into EndNote X9 for reference management and duplicate removal. Reference lists of included studies and relevant reviews were also manually screened to identify additional eligible studies.

### 2.4 Eligibility criteria

The eligibility criteria were developed using the Population Concept Context (PCC) framework (29) and are summarised in Table 1. Non-obstetric gynaecological examinations were operationally defined as intimate clinical examinations or diagnostic procedures involving visualisation, palpation, instrumentation, imaging, or exposure of the genital or pelvic area. Eligible procedures included routine pelvic examination, speculum examination, bimanual examination, colposcopy, transvaginal ultrasound, and other diagnostic procedures involving direct genital or pelvic exposure. General gynaecological consultations, contraceptive or fertility counselling, and treatment-focused surgical procedures were excluded unless separately identifiable examination-related experiences were reported. Where studies included mixed participant groups, findings relating specifically to women had to be separately identifiable. The concept of interest comprised communication, privacy, and interpersonal care processes related to women's examination experiences or care-related outcomes. These included information-giving, consent or permission, privacy and dignity, bodily exposure, clinician behaviour, clinician gender, chaperone or companion preferences, and the examination environment. Relevant experiences or outcomes included preparedness, perceived control, embarrassment, shame, anxiety, fear, discomfort, pain, trust, and disclosure. Follow-up attendance, delayed care, care avoidance, and willingness to return were also eligible when explicitly linked to an examination-related experience. Eligible contexts included outpatient and inpatient services, emergency departments, cervical screening and colposcopy services, transvaginal ultrasound or other imaging services, and community health services involving a non-obstetric gynaecological examination or related intimate procedure. Post-examination communication and follow-up were eligible when they formed a direct continuation of the examination pathway. Studies conducted exclusively in obstetric, intrapartum, or postpartum settings were excluded, as were studies in which non-obstetric findings could not be distinguished from obstetric findings. Quantitative, qualitative, and mixed-methods primary empirical studies, including eligible dissertations, were included if available in English or Chinese. There were no restrictions on country, publication year, or study design. Reviews, protocols, editorials, commentaries, expert opinion papers, clinical guidelines, conference abstracts without sufficient empirical detail, and other non-empirical reports were excluded.

**Table 1:** Eligibility criteria based on the PCC framework and types of sources.

Domain	Inclusion criteria	Exclusion criteria
Population	Women undergoing, awaiting, or reporting previous experience of a non-obstetric gynaecological examination or related intimate procedure. In studies involving mixed participant groups, findings relating specifically to women had to be separately identifiable.	Studies reporting only the perspectives of clinicians, students, partners, or other non-patient groups without women's examination experiences as a central outcome; studies in which women-specific findings could not be identified separately.
Concept	Communication, privacy, and interpersonal care processes related to women's examination experiences or care-related outcomes, including information-giving, consent or permission, privacy and dignity, bodily exposure, clinician behaviour, clinician gender, chaperone or companion preferences, and the examination environment. Relevant outcomes included preparedness, perceived control, embarrassment, shame, anxiety, fear, discomfort, pain, trust, disclosure, follow-up attendance, delayed care, and care avoidance. Downstream outcomes such as disclosure, willingness to return, follow-up, delayed care, or avoidance were included only when explicitly linked to an examination-related experience.	Studies focused mainly on technical performance, diagnostic accuracy, clinical effectiveness, examination or surgical technique, or disease outcomes without reporting examination-related experiences. Studies concerned solely with general access to women's health services, overall satisfaction with gynaecology services, population-level screening uptake, or disease management without an identifiable examination-specific care process or experience.
Context	Any healthcare setting involving a non-obstetric gynaecological examination or related intimate procedure, including outpatient and inpatient services, emergency departments, cervical screening and colposcopy services, transvaginal ultrasound or other imaging services, and community health services. Post-examination communication and follow-up were eligible when they formed a direct continuation of the examination pathway.	Studies conducted exclusively in obstetric, intrapartum, or postpartum settings; studies in which non-obstetric findings could not be distinguished from obstetric findings; gynaecology attendance or cervical screening without an explicit connection to an intimate examination, related procedure, or examination-specific experience. General gynaecological consultations, contraceptive or fertility counselling, and treatment-focused procedures without separately identifiable examination-related experiences were excluded.
Types of sources	Quantitative, qualitative, and mixed-methods primary empirical studies, including eligible dissertations, available in English or Chinese, with no restriction on country, publication year, or study design.	Reviews, protocols, editorials, commentaries, expert opinion papers, clinical guidelines, conference abstracts without sufficient empirical detail, and other non-empirical reports.

### 2.5 Study selection

Study selection followed a two-stage screening process. After duplicate removal, titles and abstracts were independently screened by two reviewers against the eligibility criteria. Potentially relevant reports then underwent independent full-text assessment by the same reviewers. Records identified through the CNKI Dissertations Database were screened using the same eligibility criteria. Disagreements were resolved through discussion, with consultation of a third reviewer when consensus could not be reached. Reference lists of included studies and relevant reviews were subsequently screened to identify additional eligible studies. The study selection process is shown in Figure 1.

**Figure 1:**
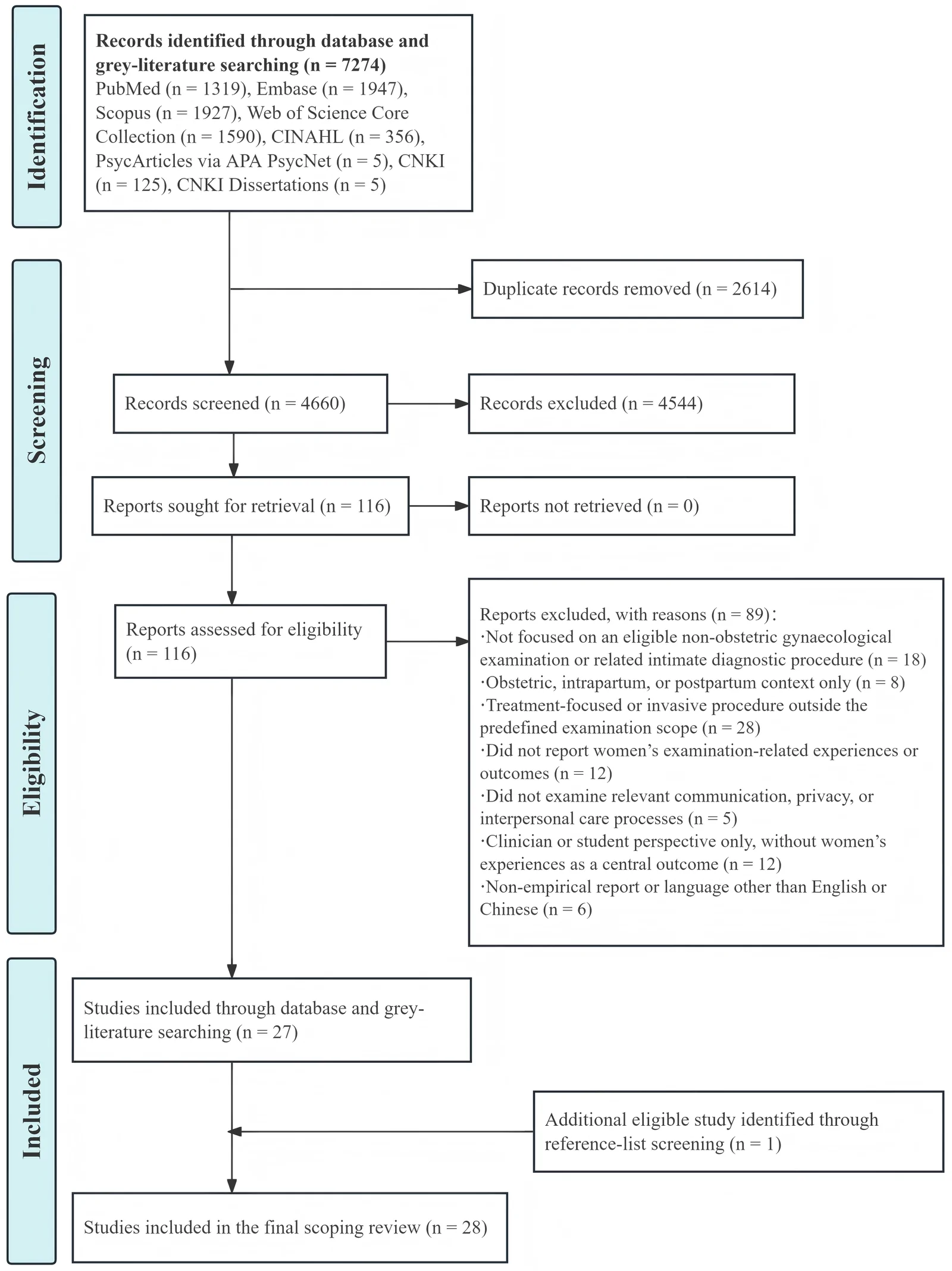
PRISMA flow diagram of study selection.

### 2.6 Data extraction

Data were independently extracted by two reviewers using a data-charting form developed in Microsoft Excel. The form was refined during the initial stage of extraction through discussion within the research team. Extracted information included author and year, country, study design, participant characteristics, sample size, examination context or procedure, communication and care-process factors, reported experiences or outcomes, and key findings relevant to the review objectives. Differences in data extraction or interpretation were resolved through discussion and consensus.

### 2.7 Data synthesis and evidence mapping

Because the included studies varied in design, examination context, participant characteristics, and outcome measures, quantitative findings were summarised descriptively and no statistical pooling was undertaken. Quantitative findings were charted as reported prevalence estimates, associations, group differences, or changes in examination-related experiences and outcomes, while qualitative findings were charted from the themes, categories, and interpretive findings reported in the original primary studies. Integration of quantitative and qualitative evidence occurred at the level of the reported care-process factor and associated experience or outcome rather than through conversion to a common effect measure. Within the collation and synthesis stage, thematic evidence mapping was used to identify recurring communication, privacy, and interpersonal care processes and their associated experiences or outcomes, with the initial coding framework informed by the thematic analysis principles described by Braun and Clarke (30); this approach was adopted to synthesise diverse findings across quantitative and qualitative study designs, and is distinct from thematic analysis conducted on primary raw data. Where findings were inconsistent or contradictory across studies, such as the effect of pre-examination information on anxiety or the magnitude of clinician gender preferences, no attempt was made to reconcile, aggregate, or average the findings; instead, heterogeneity was explicitly presented in the Results section, and potential sources of variation including differences in examination context, study population, outcome measurement instruments and study design were addressed in the Discussion section. Studies were initially grouped by examination context to preserve clinically relevant differences between procedures, and initial codes were generated from the extracted care-process factors, reported experiences or outcomes, and key findings, with codes of similar meaning compared, merged, or refined through discussion between the reviewers. During the final stage of synthesis, a study-level evidence-mapping matrix was constructed from the extracted data to organise examination context, study design, communication or care-process factors, reported experiences or outcomes, encounter stage, and relevant cross-cutting contextual influences. One reviewer completed the initial mapping, and the second reviewer checked the classifications against the extracted findings and source studies, with disagreements concerning coding or stage allocation resolved through discussion and consensus. Findings were mapped according to whether they related to the pre-examination, intra-examination, or post-examination period, and studies could contribute to more than one stage when they reported findings relating to different parts of the encounter. After-examination findings were further distinguished between immediate responses and less frequently reported findings concerning subsequent care engagement, and factors reported across more than one stage were classified as cross-cutting contextual influences. The stage-based evidence map was developed iteratively from the coded findings rather than imposed as a fixed explanatory or causal model in advance, and the research team reviewed stage allocation, cross-cutting categories, and the overall structure against the extracted evidence. The resulting evidence map is presented in the Results section, and is intended to organise the available literature rather than imply procedural equivalence, a fixed sequence, or causal relationships.

### 2.8 Methodological quality appraisal

No formal methodological quality appraisal was undertaken because the purpose of this scoping review was to map the range, characteristics, and gaps of the available evidence rather than to determine certainty of effect. Studies were therefore not excluded or weighted according to methodological quality.

## 3 Results

### 3.1 Study selection

A total of 7274 records were identified through the seven electronic databases and the CNKI Dissertations Database: PubMed (*n* = 1,319), Embase (*n* = 1,947), Scopus (*n* = 1,927), Web of Science Core Collection (*n* = 1,590), CINAHL (*n* = 356), PsycArticles via APA PsycNet (*n* = 5), CNKI (*n* = 125), and CNKI Dissertations (*n* = 5). After removal of 2,614 duplicate records, 4,660 records remained for title and abstract screening, of which 4,544 were excluded. All 116 reports sought for retrieval were obtained and assessed for eligibility, of which 89 were excluded with reasons summarised in the PRISMA flow diagram (Figure 1): not focused on an eligible non-obstetric gynaecological examination or related intimate diagnostic procedure (*n* = 18); exclusive obstetric, intrapartum, or postpartum context (*n* = 8); treatment-focused or invasive procedures outside the predefined examination scope (*n* = 28); no report of women's examination-related experiences or outcomes (*n* = 12); no examination of relevant communication, privacy, or interpersonal care processes (*n* = 5); only clinician or student perspectives without women's experiences as a central outcome (*n* = 12); and non-empirical reports or publications in languages other than English or Chinese (*n* = 6). Twenty-seven studies met the inclusion criteria through the database and grey-literature searches. One additional eligible study was identified through reference-list screening. In total, 28 studies were included in the review. The study-selection process is presented in Figure 1.

### 3.2 Characteristics of included studies

Twenty-eight studies published between 1993 and 2026 were included, comprising 20 quantitative and eight qualitative studies. Quantitative studies were predominantly cross-sectional, descriptive, correlational, or comparative, with a smaller number of prospective and intervention studies. Qualitative studies primarily used individual interviews or focus groups to examine women's experiences and perceptions of gynaecological examinations. Women-specific sample sizes ranged from six participants in a qualitative interview study to 6,508 participants in a large electronic survey. The studies were conducted in 11 countries: Turkey (*n* = 7), the United States (*n* = 7), Denmark (*n* = 2), Poland (*n* = 2), Sweden (*n* = 2), Nigeria (*n* = 2), China (*n* = 2), Israel (*n* = 1), Iran (*n* = 1), Brazil (*n* = 1), and Singapore (*n* = 1). Examination contexts included routine gynaecological or pelvic examinations, vaginal and speculum examinations, colposcopy and cervical-screening follow-up, transvaginal ultrasound, emergency-department pelvic examinations, and examination-related gynaecological consultations. Several studies focused on specific populations or contexts, including young women undergoing their first pelvic examination, women with low incomes, and women with abnormal cervical-screening results, women who had missed colposcopy follow-up, Deaf women, and women undergoing examination by male clinicians. Where studies also included physicians, partners, or other participant groups, women-specific findings were extracted and prioritised. Detailed study characteristics and findings are presented in Table 2.

**Table 2:** Characteristics and key findings of the included studie**s.**

Study	Author, year	Country	Study design	Participants and sample size	Examination context or procedure	key findings relevant to the review objectives
No.1	Laschke et al., 2023 (31)	Denmark	Quantitative	Women attending six specialist gynaecology practices (*n* = 2,917)	Routine gynaecological examination in specialist outpatient practice, including speculum examination and bimanual palpation	Privacy and modesty preferences included a private changing area (37%), a covering garment (20%), and separate consultation and examination rooms (18%). Women outside the workforce more often reported inadequate information, unprofessional care, and pain.
No.2	Bryan and Chor, 2018 (32)	USA	Qualitative	Women aged 18–24 years who had undergone at least one pelvic examination (*n* = 30)	First pelvic examination including speculum insertion and bimanual palpation	Preparedness was reported in relation to age at the first examination, prior knowledge, medical trust or mistrust, comfort with one's body, and previous sexual experiences or trauma. Participants also described pre-examination information in relation to preparedness.
No.3	Hacker et al., 2022 (33)	USA	Qualitative	Cisgender women with low incomes who had experience of pelvic examinations (*n* = 12)	Routine pelvic examination in primary care, including speculum and bimanual examination	Four themes were identified: objectification, a cycle of uncertainty, negotiation of agency, and the potential for positive experiences. Rapport, respectful communication, choice, and continuity of care were described in accounts of more acceptable encounters.
No.4	Bay and Akin, 2022 (34)	Turkey	Quantitative	Women who had undergone a pelvic examination (*n* = 415)	Pelvic examination including speculum examination and bimanual palpation	Body-privacy scores differed according to whether women had received information before the examination, whereas state-anxiety scores did not differ significantly by pre-examination information status.
No.5	Tancman et al., 2022 (35)	Israel	Quantitative	Women with experience of at least one gynaecological examination (*n* = 6,508)	Gynaecological examination including pelvic inspection, speculum and bimanual palpation	The examination was described as embarrassing by 47%, painful by 35%, and traumatic by 19%. Communication was rated as the most important aspect of the encounter; only 43% said they would tell the clinician about discomfort or pain, and 62.7% valued the option of a companion.
No.6	Yilmaz and Demirel, 2021 (36)	Turkey	Quantitative	Women presenting for a gynaecological examination (*n* = 349)	Gynaecological examination including pelvic and speculum examination	Women attached high importance to body privacy and all experienced pre-examination anxiety. Privacy-related concerns explained 16.3% of the variance in anxiety.
No.7	Yanikkerem et al., 2009 (12)	Turkey	Quantitative	Women attending a gynaecological outpatient clinic for examination (*n* = 433)	Gynaecological outpatient examination including pelvic inspection and speculum examination	Nearly half reported anxiety or worry, and 41.8% reported embarrassment associated with undressing. Women expected explanation, listening, empathy, understandable language, and respectful engagement from doctors and nurses.
No.8	Niziurski, 2016 (37)	Poland	Quantitative	Non-pregnant women surveyed in a private gynaecology office and a university setting (*n* = 331)	Gynaecological visit including pelvic examination, speculum and bimanual palpation	The examination chair and examination itself were the most embarrassing parts of the visit; vaginal and rectal palpation caused the greatest stress. Women expected the gynaecologist to be pleasant, talkative, and calm, and most felt safest alone with the doctor.
No.9	Szymoniak et al., 2009 (38)	Poland	Quantitative	Women attending two hospital gynaecology clinics (*n* = 100)	Hospital gynaecological examination including speculum and bimanual palpation	Seventy percent described the examination as embarrassing and stressful, and 47% reported that intimacy was not ensured. A female doctor was preferred by 56%, while 46% said a qualified midwife's presence increased safety and psychological comfort.
No.10	Oscarsson and Benzein, 2002 (39)	Sweden	Qualitative	Women with experience of multiple pelvic examinations (*n* = 6)	Pelvic examination in primary care including bimanual and speculum examination	Three themes were identified: being exposed, having a trusting relationship with the examiner, and experiencing a comforting environment. Trust, encouragement to ask questions, and a homelike setting were reported in more positive examination experiences.
No.11	Umeora et al., 2011 (40)	Nigeria	Quantitative	Gynaecology clinic attendees undergoing digital and speculum vaginal examinations (*n* = 486)	Digital palpation and speculum vaginal examination	Shyness or embarrassment was reported by 66.3%, and 37.2% reported pain or discomfort. Despite this, 73.7% regarded the examination as necessary and 95.9% were willing to undergo it again if required.
No.12	Aktas et al., 2018 (41)	Turkey	Quantitative	Women attending an outpatient clinic for a gynaecological complaint or routine examination (*n* = 120)	Gynaecological outpatient examination including pelvic and speculum examination	Post-examination anxiety was lower in all intervention groups than in the control group, with the lowest anxiety in women who used a special examination gown and listened to nature-based sounds.
No.13	Baser et al., 2013 (42)	Turkey	Quantitative	Women undergoing colposcopy for abnormal cervical cytology (*n* = 222)	Colposcopy after abnormal cervical cytology, involving speculum placement and cervical visualisation	Higher pre-procedural state anxiety was associated with greater colposcopy-related pain and discomfort. Higher educational attainment was associated with less discomfort.
No.14	Yaman et al., 2026 (43)	Turkey	Quantitative	Women with ASC-US or LSIL results (*n* = 94) and women with negative smear results (*n* = 94); total *n* = 188	Cervical smear results communication and follow-up, linked to prior speculum and pelvic examination	Women with low-grade abnormal smear results had significantly higher anxiety than controls. Misinterpretation of results and inadequate knowledge were identified as relevant concerns.
No.15	Ulker and Kivrak, 2016 (44)	Turkey	Quantitative	Women attending a university gynaecology clinic with minor symptoms or for routine screening (*n* = 271; control *n* = 88, examination-information group *n* = 91, comparison-information group *n* = 92)	Pre-examination information for routine gynaecological pelvic examination	Brief written information about the examination procedure and clinic workflow did not significantly reduce state or trait anxiety compared with control information or usual care.
No.16	Fathnezhad-Kazemi et al., 2025 (45)	Iran	Quantitative	Women undergoing vaginal examination during a gynaecological visit (*n* = 270)	Vaginal examination during a gynaecological visit, including speculum and digital palpation	Shame or embarrassment was reported by 74.1%, stress by nearly 60%, and pain by more than 55%. Privacy scores were positively associated with situational anxiety; shame was also reported as a reason for delayed attendance.
No.17	Wendt et al., 2004 (11)	Sweden	Qualitative	Women aged 18–82 years with experience of gynaecological examinations (*n* = 30)	Gynaecological examination including pelvic, speculum and bimanual examination	Women's accounts were organised around trust and confirmation. Participation, information, support, respect, and engagement were reported in accounts characterised by trust and confirmation, whereas accounts lacking these features were described more negatively.
No.18	Aguilar et al., 2026 (46)	USA	Qualitative	Women who had missed at least one scheduled colposcopy appointment after abnormal cervical screening (*n* = 20)	Colposcopy examination follow-up after abnormal cervical screening (involving speculum placement, cervical visualisation and diagnostic sampling)	Barriers occurred at individual, interpersonal, clinic, and system levels, including fear of pain or pelvic examination, communication of results, trainee presence and limited continuity, scheduling difficulties, competing demands, and loss of insurance coverage.
No.19	Adams et al., 1993 (47)	USA	Quantitative	Women (*n* = 291) and physicians (*n* = 1,316)	Routine pelvic examination (speculum+bimanual) frequency and attendance	As body weight increased, negative body-related attitudes and reluctance to undergo pelvic examinations increased, while annual examination frequency decreased.
No.20	Silva de Freitas et al., 2025 (10)	Brazil	Qualitative	Deaf university women (*n* = 8)	Cervical screening pathway including routine pelvic examination, speculum examination and cervical smear collection	Participants reported communication barriers, absence of professional interpreters, and reliance on a third person as an intermediary, together with difficulties involving communication, privacy, and accessibility.
No.21	Akintomide and Obasi, 2019 (48)	Nigeria	Quantitative	Women referred for transvaginal ultrasound (*n* = 204)	Transvaginal ultrasound, involving transvaginal probe insertion and pelvic imaging	Awareness was low (20.6%) but acceptance was high (98.5%). Forty-six percent had no preference for sonologist gender, 45% preferred a woman, and about half wanted a third person present, most often their husband.
No.22	Moettus et al., 1999 (49)	USA	Quantitative	Female emergency-department patients undergoing pelvic examination (*n* = 167)	Emergency-department pelvic examination including speculum and bimanual assessment	Examinations by male clinicians were rated as significantly more embarrassing than examinations by female clinicians, but perceived pain did not differ significantly by clinician gender.
No.23	Patton et al., 2003 (50)	USA	Quantitative	Emergency-department patients undergoing internal pelvic examination (*n* = 278)	Emergency-department internal pelvic examination including digital and speculum assessment	Clinician gender and level of training did not predict pain or embarrassment. Most women (62%) had no gender preference; 34% preferred a woman and 4% preferred a man.
No.24	Chen and Yang, 2008 (51)	China	Quantitative	Female gynaecology outpatients examined by male doctors (*n* = 113) or female doctors (*n* = 137); total *n* = 250	Pelvic examination including speculum and bimanual palpation, by male vs. female clinicians	Eighty percent were unwilling or strongly unwilling to be examined by a male doctor. Women examined by male doctors reported significantly more nervousness, fear, shame, uneasiness, and humiliation than those examined by female doctors.
No.25	Li et al., 2021 (52)	China	Qualitative	Women (*n* = 55) and male partners (*n* = 21); total *n* = 76	Routine gynaecological examination including pelvic and speculum examination	Among women, 43.64% described embarrassment, nervousness, or fear and 18.18% reported a previous poor examination experience. The study also reported preferences for more humane services, stronger clinician–patient communication, and partner support.
No.26	Hilden et al., 2003 (53)	Denmark	Quantitative	Consecutive women attending a hospital department of obstetrics and gynaecology who completed a questionnaire concerning the index visit (*n* = 798)	Gynaecological examination during an outpatient hospital visit, including speculum and bimanual palpation	Discomfort during the examination was associated with negative emotional contact with the examiner and younger age. A history of sexual abuse, mental health problems, and dissatisfaction with current sexual life were also associated with discomfort.
No.27	Davenport et al., 2016 (54)	USA	Quantitative	Adult women scheduled for transvaginal pelvic ultrasound at three outpatient centres and one inpatient centre in a single health system (*n* = 155)	Transvaginal pelvic sonography, involving transvaginal probe insertion	Chaperone preferences depended strongly on sonographer gender. Before the examination, 46% preferred a chaperone if the sonographer was male, compared with 12% if the sonographer was female; after the examination, the corresponding proportions were 43% and 6%. Anxiety decreased from 22% before to 8% after the examination, while chaperone preferences changed little.
No.28	Lee et al., 2024 (55)	Singapore	Qualitative	Women aged 30–57 years who had undergone a vaginal examination or transvaginal ultrasound within the previous year (*n* = 12)	Outpatient vaginal examination (speculum+digital) and transvaginal ultrasound	Privacy, stepwise information, gentle and responsive care, warm lubricant, and a calm environment were described in accounts of greater comfort and perceived control; rushed or rough care and inadequate communication were reported in accounts of unpleasant experiences.

ASC-US, atypical squamous cells of undetermined significance; LSIL, low-grade squamous intraepithelial lesion. Study numbers correspond to the order in which the studies are presented in the table. For studies that also included physicians, partners, or other participant groups, findings specific to women were extracted and prioritised; non-patient findings were retained only where directly relevant to women’s examination experiences or access to care.

### 3.3 Findings across stages of the encounter

Findings were organised according to whether the reported communication, privacy, and interpersonal care processes occurred in the pre-examination, intra-examination, or post-examination phase. Studies could contribute to more than one stage when findings concerned different parts of the examination encounter. Post-examination findings were further separated into immediate responses and findings concerning subsequent care engagement. Cross-cutting contextual influences reported across more than one stage included previous examination experiences, body image, socioeconomic circumstances, communication barriers, and concerns related to diagnosis or follow-up. Table 3 presents the contributing studies by encounter stage, and Figure 2 provides a stage-based evidence map of the included findings.

**Table 3:** Evidence map by stage of the gynaecological examination encounter

Encounter stage	Communication and care-process factors	Reported experiences or outcomes	Studies^a^
Pre-examination phase	Prior knowledge and previous examination experience; procedural information and explanation; expectations of clinicians; written information; communication of screening results and next steps; communication accessibility and interpreter support; practical barriers to attending or completing follow-up care.	Preparedness, anticipatory anxiety, uncertainty, understanding, and readiness for examination or subsequent care.	No.1, 2, 4, 5, 7, 11, 14, 15, 18, 20, 27, 28
Intra-examination phase	Privacy and management of bodily exposure; changing and examination environments; draping or covering; explanation during the procedure; perceived control and agency; clinician gentleness, respect, responsiveness, rapport and emotional contact; clinician gender; chaperone, assistant or companion preferences.	Embarrassment, fear, pain, discomfort, objectification, vulnerability, perceived privacy, trust, confirmation, perceived control, and emotional comfort or safety.	No.1, 3, 5–10, 12,16,17, 20–28
Post-examination phase: immediate responses	Women's appraisal of the examination, bodily exposure, procedure, clinician conduct, and examination technique.	Embarrassment, shame, anxiety, fear, pain, discomfort, stress, unpleasantness, and self-rated traumatic experience.	No.1,4,5,7–9,11–13,16,22–28
Post-examination phase: subsequent care engagement	Disclosure of pain or discomfort; perceived safety in returning; willingness to undergo another examination; follow-up attendance; delayed care; reluctance or avoidance; examination frequency and continuity of care.	Disclosure or non-disclosure, willingness to return or repeat examination, expressed reluctance to attend, delayed attendance, missed follow-up, reduced examination frequency, and avoidance of subsequent care.	No.1,5,11,16,18–20,28

a Study numbers correspond to Table 2. Some studies contributed to more than one stage of the encounter. Stage allocation was based on the timing and content of findings reported in the included studies and does not imply that the examinations or procedures were clinically equivalent or that the stages represent a causal sequence. Fewer studies reported findings concerning subsequent care engagement than immediate examination-related responses.

**Figure 2:**
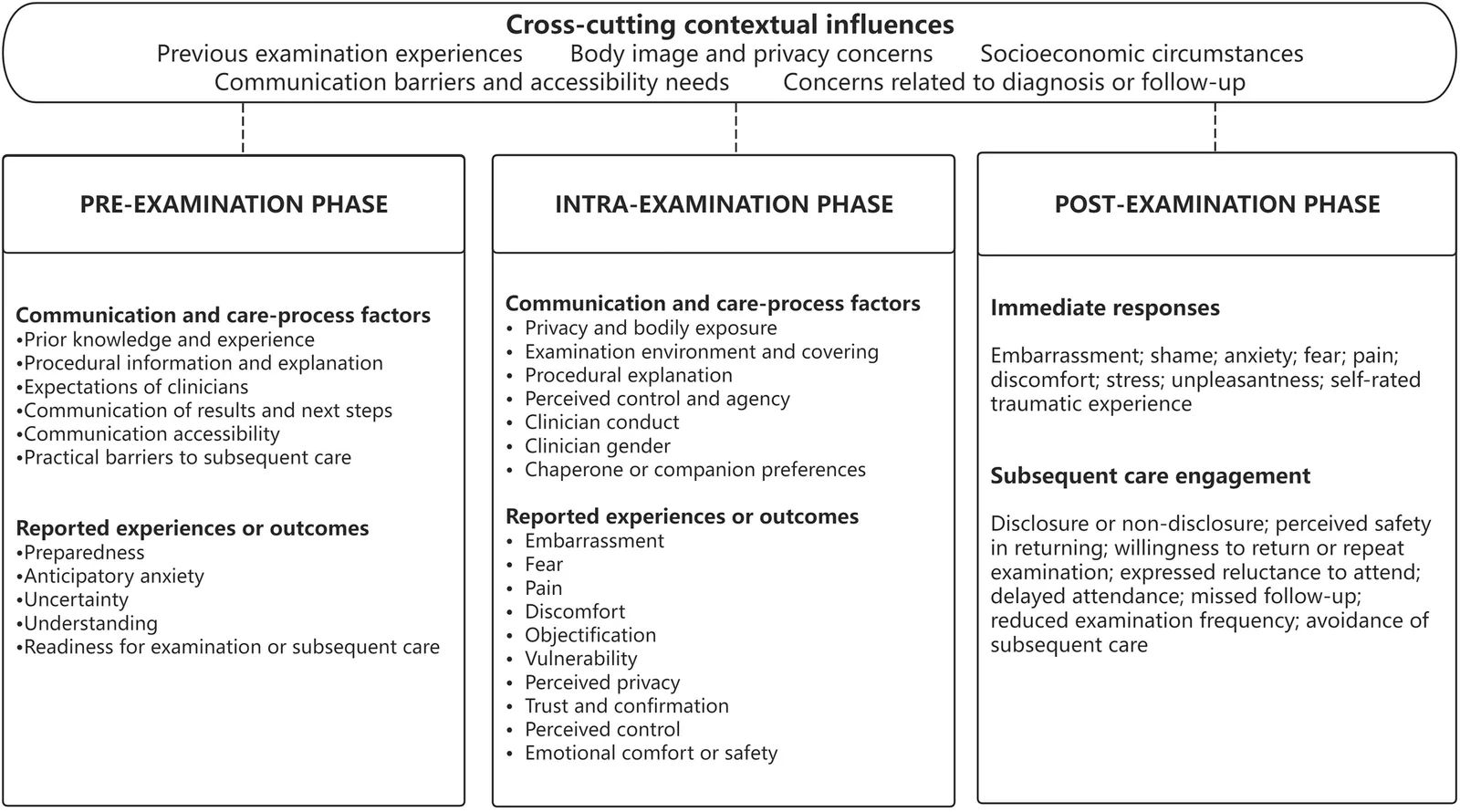
Stage-based evidence map of communication, privacy, and interpersonal care processes across the gynaecological examination encounter. Findings are organised according to where communication, privacy, and interpersonal care processes were reported across the pre-examination, intra-examination, and post-examination phases. Cross-cutting contextual influences were reported across more than one phase. The post-examination phase is further divided into immediate responses and subsequent care engagement. The arrangement is intended for descriptive evidence mapping and does not imply procedural equivalence, a fixed sequence, or causal relationships.

### 3.4 Pre-examination phase: preparation, information, expectations, and readiness for care

In the pre-examination phase, the evidence concerned prior knowledge and experience, procedural information, expectations of clinicians, anticipatory anxiety, communication accessibility, and preparation for subsequent care. In sequential screening or follow-up pathways, the pre-examination phase referred to preparation for the next eligible intimate examination or follow-up procedure; relevant communication could therefore occur after an earlier screening test. Across studies, women commonly described wanting to know why an examination was required, what would happen, what discomfort might occur, and what would happen afterwards (Study Nos. 2, 5, 7, 18, 20, and 28). Preparedness was also shaped by previous examination experiences, trust or mistrust in healthcare, comfort with one's body, and previous sexual experiences or trauma (Study No. 2). Several quantitative studies indicated that information was highly valued, although its relationship with anxiety was inconsistent. In a survey of 2,917 women attending specialist gynaecology practices (Study No. 1), 93.7% reported receiving a thorough explanation of the examination, while women not in paid employment were more likely to report insufficient information (OR 1.40, 95% CI 1.11–1.75). In a larger survey of 6,508 women (Study No. 5), more than 97% rated explanations before and during the examination, warnings before potentially painful procedures, and time for questions as important or very important. However, reported receipt of these elements was lower: 24.1% reported usually or always receiving satisfactory explanations, 36.0% reported being warned before painful procedures, and 53.7% reported usually or always having time for questions after examination. Among 415 women undergoing pelvic examination (Study No. 4), pre-examination information was associated with differences in body-privacy scores but not with state anxiety. Similarly, a prospective randomised study (Study No. 15) found that brief written information about the examination did not significantly reduce state or trait anxiety compared with control conditions.

Studies concerning cervical screening, colposcopy, and transvaginal ultrasound highlighted additional informational and practical needs. Women with low-grade abnormal cervical-smear results reported greater anxiety than women with negative results, while previous knowledge and prior screening experience were relevant within the abnormal-result group (Study No. 14). Among women who had missed colposcopy follow-up (Study No. 18), barriers included fear of pain or pelvic examination, difficulty obtaining answers to questions, scheduling problems, competing responsibilities, other health conditions, and insurance-related difficulties. Deaf women (Study No. 20) described lack of professional interpreters, reliance on companions, and difficulty understanding examination procedures. Before transvaginal ultrasound, anxiety and uncertainty were also reported (Study No. 27), particularly when the examination was unfamiliar. Qualitative accounts similarly emphasised the value of information about examination steps, duration, and possible discomfort (Study No. 28). Taken together, the evidence suggests that preparation involves more than the provision of procedural facts. Women's readiness for examination was reported in relation to prior experience, expectations, communication accessibility, and opportunities to understand and anticipate what would occur. However, the available evidence does not establish that information provision alone consistently reduces anxiety.

### 3.5 Intra-examination phase: privacy, control, interpersonal conduct, and support preferences

In the intra-examination phase, the evidence centred on privacy and management of bodily exposure, the physical environment, explanation and perceived control, clinician conduct, clinician gender, and preferences regarding chaperones or companions. Privacy-related concerns were prominent across both quantitative and qualitative studies (Study Nos. 1, 5, 6, 9, 10, 16, and 28). Women reported valuing private changing areas, curtains, examination garments or coverings, and reduced bodily exposure. For example, 94.7% of women in one large survey (Study No. 5) rated a private place to undress as important or very important, 88.3% valued a curtain around the examination bed, and 87.7% valued provision of a body covering. Privacy-related measures were also associated with anxiety in two quantitative studies (Study Nos. 6 and 16), although different instruments and study designs limit direct comparison.

Qualitative findings extended privacy beyond the physical environment. Women described bodily exposure in relation to trust, objectification, agency, participation, and perceived control (Study Nos. 3, 10, 17, and 28). More acceptable encounters were characterised by respectful communication, support, reassurance, and the ability to understand or influence how the examination proceeded. In contrast, uncertainty, inadequate communication, and negative interpersonal rapport were associated with less acceptable experiences. In one quantitative study of 798 women (Study No. 26), negative emotional contact with the examiner was strongly associated with examination discomfort (OR 8.21, 95% CI 3.91-17.24), although the cross-sectional design did not establish causality.

Communication and responsiveness during examination were also represented across clinical settings. Women reported expectations that clinicians would be understanding, gentle, communicative, and attentive to discomfort. Among 433 women attending a gynaecological clinic (Study No. 7), 55.2% expected understanding and gentle behaviour from doctors and 71.8% expected this from nurses. For Deaf women (Study No. 20), communication barriers were described alongside insecurity, nervousness, pain, fear, embarrassment, and difficulty understanding what was occurring when clinicians communicated through accompanying family members rather than directly with them.

Preferences regarding clinician gender were heterogeneous (Study Nos. 9, 21-24, and 26). Some studies reported strong preferences for female clinicians or greater embarrassment during examination by male clinicians (Study Nos. 9, 22, and 24). In one Chinese study (Study No. 24), 80% of women reported being unwilling or strongly unwilling to undergo pelvic examination by a male doctor, and women examined by male doctors reported greater nervousness, fear, shame, uneasiness, and humiliation. In contrast, other studies found no significant association between clinician gender and pain, embarrassment, or discomfort (Study Nos. 23 and 26), and in one emergency-department study (Study No. 23) 62% of women reported no gender preference. Preferences regarding third-person presence were similarly variable (Study Nos. 1, 5, 9, 21, and 27). In one Danish study (Study No. 1), 13.0% considered another person's presence important or very important, whereas 62.4% of women in another survey (Study No. 5) valued the option of a companion. Among women undergoing transvaginal ultrasound (Study No. 27), chaperone preferences varied according to sonographer gender: before examination, 46% preferred a chaperone when the sonographer was male compared with 12% when the sonographer was female. Overall, the findings indicate that privacy, perceived control, interpersonal conduct, clinician gender, and third-person presence are interrelated features of the examination encounter, but individual preferences vary substantially and should not be assumed to be uniform.

### 3.6 Post-examination phase: immediate responses

Studies reporting immediate examination-related responses predominantly assessed embarrassment, shame, anxiety, fear, pain, discomfort, stress, or unpleasantness (Study Nos. 1, 4, 5, 7-9, 11-13, 16, and 22-28). Across clinical settings, these responses were common, although their frequency and measurement varied considerably. In one survey of 6,508 women (Study No. 5), 47.1% described the examination as embarrassing or very embarrassing, 35.6% as physically uncomfortable or painful, and 18.6% as traumatic or highly traumatic; the latter reflected participants' self-ratings rather than a clinical assessment of trauma. Several other studies also reported substantial levels of embarrassment or shame, although estimates varied across examination contexts and measures (Study Nos. 7, 9, 11, 16, and 25).

The specific sources of distress also varied. Undressing, genital exposure, positioning in the gynaecological chair, examination materials, and fear of pain were repeatedly identified as unpleasant or stressful aspects of the encounter (Study Nos. 7-9 and 16). Among women undergoing colposcopy (Study No. 13), higher pre-procedural state anxiety was significantly associated with greater procedure-related pain and discomfort. Conversely, some intervention evidence suggested that aspects of the examination environment may influence immediate anxiety: one study (Study No. 12) reported lower post-examination state-anxiety scores among women receiving a special examination gown, nature-based sounds, or both compared with usual care. Clinician and procedural characteristics did not show consistent associations with immediate responses. In one emergency-department study (Study No. 22), examinations by male clinicians were rated as more embarrassing than those by female clinicians, whereas pain did not differ significantly by clinician gender; another emergency-department study (Study No. 23) found no significant association between practitioner gender and either pain or embarrassment. Anxiety also decreased after transvaginal ultrasound in one prospective study (Study No. 27), from 22% before examination to 8% afterwards. Qualitative accounts further showed that rushed or rough examination and inadequate communication could contribute to unpleasant experiences, whereas gentleness, explanation, and responsiveness were described in more acceptable encounters (Study No. 28).

### 3.7 Post-examination phase: subsequent care engagement

Fewer studies examined what occurred after the immediate examination experience, including disclosure of discomfort, willingness to return or undergo another examination, delayed attendance, missed follow-up, reluctance, and avoidance (Study Nos. 1, 5, 11, 16, 18-20, and 28). The available findings were heterogeneous and did not support a single pathway from a difficult examination experience to subsequent care behaviour.

Disclosure of discomfort was not universal. In one large survey (Study No. 5), only 43.4% of women stated that they would report physical discomfort or pain to the gynaecologist. Among women who had not attended a gynaecological examination during the previous year, reported reasons included perceived lack of need, physical discomfort, difficulty finding a trusted gynaecologist, being busy or forgetting, a previous examination described as traumatic, and previous sexual trauma. Among women who had changed gynaecologists, discomfort with the previous clinician and unanswered questions were also reported. At the same time, negative immediate experiences did not necessarily imply unwillingness to return. In one Danish study (Study No. 1), 98.1% of women reported feeling safe returning to a gynaecologist after examination, and 95.9% of women in a Nigerian study (Study No. 11) stated that they would undergo another vaginal examination if required despite commonly reported embarrassment and discomfort. These findings suggest that immediate distress and subsequent engagement should be treated as related but distinct outcomes. Delayed attendance and missed follow-up were reported in a smaller number of studies and were associated with multiple factors (Study Nos. 16 and 18). Reasons included financial difficulty, shame, fear of serious illness, fear of pain or pelvic examination, competing work or family responsibilities, communication problems, lack of continuity, scheduling difficulties, and insurance-related barriers. Among women who had missed colposcopy appointments (Study No. 18), reminders, direct clinic communication, and family support were described in accounts of eventual attendance. Other studies reported reluctance to undergo pelvic examinations (Study No. 19), communication-related avoidance among Deaf women (Study No. 20), or individual accounts of prolonged non-attendance following a previous examination described as rough (Study No. 28). Overall, evidence concerning subsequent care engagement was substantially less developed than evidence concerning immediate emotional and physical responses. Direct links between a specific examination experience and later attendance or avoidance were uncommon, and structural, practical, informational, interpersonal, and psychosocial factors often co-occurred. The existing literature therefore supports framing subsequent care engagement as a key evidence gap, rather than inferring a consistent causal relationship between examination experience and subsequent healthcare utilisation.

## 4 Discussion

This scoping review mapped communication, privacy, and interpersonal care processes across non-obstetric gynaecological examinations and related intimate diagnostic procedures. The 28 included studies indicate that these processes extend across the examination encounter rather than being confined to the procedure itself. Evidence from the pre-examination phase focused on preparation, information, expectations, and communication accessibility; evidence from the intra-examination phase centred on privacy, bodily exposure, communication, perceived control, clinician conduct, clinician gender, and third-person presence. By contrast, subsequent disclosure, return for care, delayed attendance, follow-up, and avoidance were examined much less frequently. The high prevalence of embarrassment, anxiety and discomfort reported across included studies aligns with findings from a recent comprehensive review by Butureanu et al. (56), which characterised embarrassment as a key cross-setting barrier in gynaecological care and outlined its implications for patient engagement and service utilisation. However, substantial heterogeneity in examination context, timing of assessment, and outcome measurement across the included studies limits direct comparison of prevalence estimates. Whereas the recent systematic review by Mignot et al. (21) evaluated interventions intended to improve women's experiences of pelvic examinations, the present review maps the broader distribution of potentially modifiable care processes across routine examination contexts and encounter stages. Its key contribution is therefore not to propose a causal pathway, but to identify where communication, privacy, and interpersonal care have received empirical attention and where clinically relevant evidence gaps remain.

Communication emerged as a recurrent component across stages of care. Women reported wanting to understand why an examination was required, what would happen, what discomfort might occur, and what would happen next (10, 12, 32, 35, 46, 55). Explanation during the procedure, opportunities to ask questions, and responsiveness to pain or uncertainty were also represented in the included evidence (11, 33, 35, 39, 55). These findings are consistent with broader patient–clinician communication literature linking communication with understanding, participation, trust, and the expression of concerns (7–9). However, the review also indicates that information provision should not be treated as equivalent to effective communication. Brief written information did not reduce anxiety in the randomised study by Ulker and Kivrak (44), whereas qualitative studies described communication as an ongoing interpersonal process involving preparation, stepwise explanation, opportunities for questions, and responsiveness during examination (11, 32, 33, 55). The current evidence does not establish which communication approach is most effective; however, it suggests that examination-related communication is better understood as an interactional process than as information delivery alone.

Privacy was similarly multidimensional. Included studies reported preferences for private changing areas, curtains, examination garments, and reduced bodily exposure (31, 35, 38, 39, 41, 55), while privacy-related measures were associated with anxiety in two quantitative studies (36, 45). Qualitative accounts extended this concept beyond the physical environment by linking privacy with the way exposure was managed, how clinicians communicated, whether discomfort was recognised, and whether women retained some influence over how the examination proceeded (11, 33, 39, 55). Privacy therefore appeared to involve both environmental and interpersonal dimensions. This interpretation is consistent with broader research in women's healthcare that has treated perceived privacy as a distinct component of care experience (57), although differences in study design and measurement prevent conclusions about the relative importance of specific privacy measures.

Perceived control, participation, and interpersonal conduct were less consistently measured but remained important across the evidence. Agency, choice, participation, and the ability to communicate discomfort were described in several studies (11, 33, 35, 55), and these findings overlap with recommendations for explicit consent during intimate examinations (58) and with trauma-informed principles of explanation, choice, and control (59). Trauma-informed care itself was not a construct explicitly mapped in this review, and these observed parallels should therefore not be interpreted as evidence that the included studies evaluated trauma-informed practice. Interpersonal conduct was more consistently represented: gentle, respectful, attentive, and responsive behaviour was described in more acceptable encounters, whereas rushed or rough examination, negative emotional contact, and poor communication appeared in less acceptable accounts (11, 12, 33, 35, 39, 53, 55). These processes are not restricted to the clinician performing the examination. Preparation, communication support, privacy protection, recognition of distress, and continuity may involve different members of the clinical team, including physicians, nurses, midwives, sonographers, and other professionals according to the examination context (10–12). This supports the view that interpersonal care is an attribute of the broader examination scenario, rather than just an attribute of the interaction between the examiner and the patient.

Preferences regarding clinician gender and third-person presence varied substantially. Some studies reported strong preferences for female clinicians or greater embarrassment during examination by male clinicians (38, 49, 51), while others found no significant association between clinician gender and pain, embarrassment, or discomfort, or reported that most women had no gender preference (50, 53). Similar questions about clinician-gender preferences have also been examined in broader gynaecological and urogynecological care contexts (60, 61), suggesting that such preferences are not confined to the intimate examination itself. Chaperone and companion preferences were similarly inconsistent across clinical settings (31, 35, 38, 48, 54). These differences should not be reduced to cultural explanations because the included studies were unevenly distributed across countries, populations, and healthcare contexts. Instead, the evidence supports an individualised approach in which preferences regarding clinician gender, chaperones, companions, information, and participation are discussed rather than assumed.

The review also identified contextual influences that may shape examination experiences, including previous negative experiences, younger age, body-related concerns, socioeconomic circumstances, communication barriers, mental health problems, and histories of sexual abuse or trauma (10, 31–33, 36, 45, 47, 53, 55). These factors were inconsistently measured and should not be interpreted as fixed markers of vulnerability. Rather, they indicate that women encounter intimate examinations within different personal, relational, and healthcare circumstances. The findings among Deaf women are particularly illustrative: lack of professional interpreters and reliance on companions were reported alongside difficulties involving understanding, privacy, and participation (10). This suggests that communication accessibility may intersect with other dimensions of examination experience rather than operating as an isolated service issue.

Evidence concerning subsequent care engagement was considerably weaker than evidence concerning immediate emotional and physical responses. Studies reported disclosure or non-disclosure of discomfort, willingness to return, delayed attendance, missed colposcopy follow-up, reluctance, and examination frequency (10, 31, 35, 40, 45–47, 55), but these outcomes represented distinct aspects of engagement rather than stages in a common sequence. Structural and practical factors such as financial constraints, competing demands, scheduling difficulties, insurance problems, communication barriers, and perceived lack of need often co-occurred with examination-related fear or discomfort (35, 45, 46). Moreover, some women remained willing to return despite embarrassment, pain, or discomfort (31, 40). The current evidence therefore does not support the conclusion that a difficult examination experience leads directly to later avoidance. Instead, it identifies a plausible but insufficiently studied relationship between examination experience and subsequent engagement with care.

A key priority for future research is to connect immediate examination experiences with subsequent care engagement. Because most included studies were cross-sectional or descriptive, prospective and longitudinal designs are needed to examine whether communication, privacy, perceived control, and interpersonal conduct are associated with later trust, disclosure, follow-up attendance, or avoidance. Intervention studies could evaluate clearly specified care processes, such as structured procedural explanation, privacy protection, communication prompts, or approaches that allow women to pause or express discomfort, while measuring both immediate and subsequent outcomes. Greater consistency in the definition and measurement of communication, privacy, dignity, perceived control, trust, and care engagement would also improve comparison across studies. Research involving women with disabilities or communication barriers, women experiencing socioeconomic disadvantage, and women receiving care across a wider range of healthcare settings remains particularly limited.

## 5 Clinical implications

Non-obstetric gynaecological examinations can be approached as whole clinical encounters rather than isolated technical procedures. The findings indicate that communication, privacy, perceived control, and interpersonal care are relevant before, during, and after examination. A stage-based perspective may help clinicians and services anticipate women's informational, relational, and privacy needs throughout the encounter. Before examination, this may include clear preparation and opportunities to identify concerns, communication needs, and individual preferences. During examination, stepwise explanation, careful management of bodily exposure, opportunities to express discomfort or pause, and attention to verbal and non-verbal distress are consistent with care processes represented in the included evidence. After examination, clarification of findings and next steps may support continuity when further investigation or follow-up is required. Implementation of these processes may involve different members of the clinical team according to the examination setting. Although relatively few included studies examined profession-specific contributions directly, available evidence indicates that physicians, nurses, midwives, sonographers, and other professionals may contribute to communication, privacy protection, recognition of discomfort, and continuity of care. Nurses and midwives may have particular opportunities to provide preparation and support when present across multiple stages of the encounter (12, 38). Preferences regarding clinician gender, chaperones, companions, information, and participation should be discussed individually rather than presumed. These implications should be interpreted as practice considerations rather than formally graded clinical recommendations. The available evidence does not establish the effectiveness of specific communication strategies, service models, or follow-up interventions. Prospective and intervention research is needed to determine whether changes in examination-related communication, privacy protection, perceived control, and continuity enhance both immediate experiences and subsequent care engagement.

## 6 Limitations

This review has several limitations. The included studies varied considerably in examination type, clinical setting, participant characteristics, study design, and outcome measurement. Although the studies were synthesised around common issues of communication, privacy, and interpersonal care, direct comparison between different examination procedures should therefore be made with caution. Some studies focused on communication of abnormal findings, follow-up, or later attendance rather than on the examination itself; these were included only when the findings remained clearly linked to an intimate examination or examination-related experience. Even so, broader barriers to follow-up or attendance cannot be attributed to the examination encounter alone. Most of the included evidence was cross-sectional or descriptive, and few studies followed women over time. This limits conclusions about whether examination experiences influence later disclosure, follow-up attendance, or avoidance. No formal methodological quality appraisal was undertaken because the purpose of the review was to map the range and characteristics of the available evidence rather than to determine certainty of effect. In addition, only studies available in English or Chinese were eligible. Only two included studies were published in Chinese, precluding meaningful comparison of findings by publication language. Chinese-language bibliographic searching was limited to CNKI, which was the principal Chinese academic database accessible through the authors' institutional library. Although CNKI provides broad coverage of Chinese academic literature, studies indexed exclusively in other Chinese databases may therefore have been missed. Grey-literature searching was limited to the CNKI Dissertations Database, and relevant unpublished or non-indexed evidence from other grey-literature sources may therefore have been missed.

## 7 Conclusion

Communication, privacy, and interpersonal care processes are relevant across multiple stages of non-obstetric gynaecological examinations and related intimate diagnostic procedures. The available evidence highlights preparation and information before examination; privacy, bodily exposure, communication, perceived control, clinician conduct, clinician gender, and third-person presence during examination; and emotional, physical, and care-related responses after examination. These findings support viewing the examination as a whole clinical encounter rather than as an isolated technical procedure and identify several potentially modifiable components of gynaecological and interdisciplinary care. The evidence base remains heterogeneous and largely observational, and findings concerning subsequent disclosure, return for care, delayed attendance, and avoidance were comparatively limited. The current literature does not support a consistent causal pathway from examination experience to later care behaviour. Future prospective and intervention research should examine both immediate examination experiences and longer-term care engagement, with clearer and more consistent measurement of communication, privacy, perceived control, dignity, and follow-up outcomes.
