# Supplementary material for: Communication, privacy and interpersonal care in non-obstetric gynaecological examinations: a scoping review
Source: Front Med (Lausanne). 2026 Oct 21;13:1992943. doi: 10.3389/fmed.2026.1992943 (PMC13733400; doi:10.3389/fmed.2026.1992943)
Supplement: Supplementary file 1 [file Datasheet1.pdf]

Supplementary Material 2

Search coverage: Database inception to 4 September 2026; no publication-year restriction. Studies available in English or Chinese were eligible.

Total records retrieved: 7,274 records across seven electronic databases and the CNKI Dissertations Database.

Database / source	Records retrieved	Search date
PubMed	1319	4 September 2026
Embase	1947	4 September 2026
Scopus	1927	4 September 2026
Web of Science Core Collection	1590	4 September 2026
CINAHL	356	4 September 2026
PsycArticles via APA PsycNet	5	4 September 2026
CNKI (journal articles)	125	4 September 2026
CNKI Dissertations (grey literature)	5	4 September 2026
Total records identified	7,274	

Pubmed

(((((((Pelvic Examination[MeSH Terms]) OR (Colposcopy[MeSH Terms]) OR (colposcop*[Title/Abstract]) OR (transvaginal ultrasound*[Title/Abstract]) OR (transvaginal ultrasonograph*[Title/Abstract]) OR (transvaginal sonograph*[Title/Abstract]) OR (vaginal exam*[Title/Abstract]) OR (internal pelvic examination*[Title/Abstract]) OR (Vaginal Examination[MeSH Terms])) OR (Speculum[MeSH Terms])) OR (Gynecology[MeSH Terms])) OR (((((((((((((pelvic examination*[Title/Abstract]) OR (pelvic exam*[Title/Abstract])) OR (gynecologic examination*[Title/Abstract])) OR (gynaecological examination*[Title/Abstract])) OR (gynecologic exam*[Title/Abstract])) OR (gynaecological exam*[Title/Abstract])) OR (vaginal examination*[Title/Abstract])) OR (speculum examination*[Title/Abstract])) OR (speculum exam*[Title/Abstract])) OR (bimanual examination*[Title/Abstract])) OR (intimate examination*[Title/Abstract])) OR (gynaecological consultation*[Title/Abstract])) OR (gynecologic consultation*[Title/Abstract]))) AND ((((((Informed Consent[MeSH Terms]) OR (Privacy[MeSH Terms])) OR (Communication[MeSH Terms])) OR (explain*[Title/Abstract]) OR (permission[Title/Abstract]) OR (modesty[Title/Abstract]) OR (rapport[Title/Abstract]) OR (perceived control[Title/Abstract]) OR (clinician gender[Title/Abstract]) OR (physician gender[Title/Abstract]) OR (examiner gender[Title/Abstract]) OR (sonographer gender[Title/Abstract]) OR (preference*[Title/Abstract]) OR (participation[Title/Abstract]) OR (Professional-Patient Relations[MeSH Terms])) OR (Patient-Centered Care[MeSH Terms])) OR ((((((((((((((((((((communication[Title/Abstract]) OR (interpersonal communication[Title/Abstract])) OR (doctor-patient communication[Title/Abstract])) OR (clinician-patient communication[Title/Abstract])) OR (provider-patient communication[Title/Abstract])) OR (informed consent[Title/Abstract])) OR (consent[Title/Abstract])) OR (privacy[Title/Abstract])) OR (body privacy[Title/Abstract])) OR (bodily

privacy[Title/Abstract])) OR (chaperone*[Title/Abstract])) OR (respect*[Title/Abstract])) OR (dignity[Title/Abstract])) OR (boundary[Title/Abstract])) OR (boundaries[Title/Abstract])) OR (continuity of care[Title/Abstract])) OR (care process*[Title/Abstract])) OR (care pathway*[Title/Abstract])) OR (waiting time*[Title/Abstract])) OR (accompaniment[Title/Abstract])) OR (support*[Title/Abstract])))) AND ((((((Trust[MeSH Terms]) OR (Disclosure[MeSH Terms])) OR (Anxiety[MeSH Terms])) OR (Emotions[MeSH Terms])) OR (Patient Acceptance of Health Care[MeSH Terms])) OR ((((((((((((((((((trust[Title/Abstract]) OR (preparedness[Title/Abstract]) OR (uncertainty[Title/Abstract]) OR (acceptab*[Title/Abstract]) OR (distrust[Title/Abstract])) OR (disclosure[Title/Abstract])) OR (self-disclosure[Title/Abstract])) OR (embarrassment[Title/Abstract])) OR (shame[Title/Abstract])) OR (discomfort[Title/Abstract])) OR (vulnerab*[Title/Abstract])) OR (anxiety[Title/Abstract])) OR (fear[Title/Abstract])) OR (pain[Title/Abstract])) OR (distress[Title/Abstract])) OR (care avoidance[Title/Abstract])) OR (healthcare avoidance[Title/Abstract])) OR (avoidance of care[Title/Abstract])) OR (reluctance[Title/Abstract])) OR (delayed attendance[Title/Abstract])) OR (delay in care[Title/Abstract])) OR (care acceptance[Title/Abstract])))) AND ((((((Ambulatory Care[MeSH Terms]) OR (Outpatients[MeSH Terms] OR Inpatients[MeSH Terms])) OR (Hospital Units[MeSH Terms])) OR (Community Health Services[MeSH Terms])) OR (Outpatient Clinics, Hospital[MeSH Terms])) OR ((((((((((((((outpatient*[Title/Abstract]) OR (clinic*[Title/Abstract])) OR (inpatient*[Title/Abstract])) OR (ward*[Title/Abstract])) OR (hospital*[Title/Abstract])) OR (hospital-based service*[Title/Abstract])) OR (community health service*[Title/Abstract])) OR (ambulatory[Title/Abstract])) OR (gynaecology clinic*[Title/Abstract])) OR (gynecology clinic*[Title/Abstract])) OR (gynaecology ward*[Title/Abstract])) OR (gynecology ward*[Title/Abstract])) OR (gynaecology department*[Title/Abstract])) OR (gynecology department*[Title/Abstract])))

Result:1319 Date:2026-9-4

Embase

Interface note: The final search displayed 2,266 results, comprising Articles (n = 1,947) and Clinical trials (n = 319). The 1,947 article records were exported and included in the PRISMA identification count.

('pelvic examination'/exp OR 'colposcopy'/exp OR 'pelvic examination*':ti,ab OR 'pelvic exam*':ti,ab OR 'gynecologic examination*':ti,ab OR 'gynaecological examination*':ti,ab OR 'gynecologic exam*':ti,ab OR 'gynaecological exam*':ti,ab OR 'vaginal examination*':ti,ab OR 'vaginal exam*':ti,ab OR 'speculum examination*':ti,ab OR 'speculum exam*':ti,ab OR 'bimanual examination*':ti,ab OR 'intimate examination*':ti,ab OR 'internal pelvic examination*':ti,ab OR 'colposcop*':ti,ab OR 'transvaginal ultrasound*':ti,ab OR 'transvaginal ultrasonograph*':ti,ab OR 'transvaginal sonograph*':ti,ab) AND (communication:ti,ab OR 'informed consent':ti,ab OR consent:ti,ab OR privacy:ti,ab OR respect*:ti,ab OR dignity:ti,ab OR chaperone*:ti,ab OR 'care process*':ti,ab OR explain*:ti,ab OR permission:ti,ab OR modesty:ti,ab OR rapport:ti,ab OR 'perceived control':ti,ab OR 'clinician gender':ti,ab OR 'physician gender':ti,ab OR 'examiner gender':ti,ab OR 'sonographer gender':ti,ab OR preference*:ti,ab OR participation:ti,ab) AND (trust:ti,ab OR disclosure:ti,ab OR embarrassment:ti,ab OR shame:ti,ab OR discomfort:ti,ab OR anxiety:ti,ab OR fear:ti,ab OR pain:ti,ab OR distress:ti,ab OR 'care avoidance':ti,ab OR preparedness:ti,ab OR uncertainty:ti,ab OR acceptab*:ti,ab)

Result:1947 Date:2026-9-4

Scopus

TITLE-ABS-KEY ("pelvic examination*" OR "pelvic exam*" OR "gynecologic examination*" OR "gynaecological examination*" OR "gynecologic exam*" OR "gynaecological exam*" OR "vaginal examination*" OR "vaginal exam*" OR "speculum examination*" OR "speculum exam*" OR "bimanual examination*" OR "intimate examination*" OR "internal pelvic examination*" OR colposcop* OR "transvaginal ultrasound*" OR "transvaginal ultrasonograph*" OR "transvaginal sonograph*") AND TITLE-ABS-KEY (communication OR

"informed consent" OR consent OR privacy OR chaperone* OR respect* OR dignity OR "care process*" OR explain* OR permission OR modesty OR rapport OR "perceived control" OR "clinician gender" OR "physician gender" OR "examiner gender" OR "sonographer gender" OR preference* OR participation) AND TITLE-ABS-KEY (trust OR disclosure OR embarrassment OR shame OR discomfort OR anxiety OR fear OR pain OR distress OR "care avoidance" OR preparedness OR uncertainty OR acceptab*)

Result:1927 Date:2026-9-4

WOS Core Collection

TS=("Pelvic Examination" OR "pelvic examination*" OR "pelvic exam*" OR "Vaginal Examination" OR "vaginal examination*" OR "vaginal exam*" OR "gynecologic examination*" OR "gynaecological examination*" OR "gynecologic exam*" OR "gynaecological exam*" OR "speculum examination*" OR "speculum exam*" OR "bimanual examination*" OR "intimate examination*" OR "internal pelvic examination*" OR colposcop* OR "transvaginal ultrasound*" OR "transvaginal ultrasonograph*" OR "transvaginal sonograph*" OR Speculum OR Gynecology) AND TS=("Informed Consent" OR Privacy OR Communication OR "Professional-Patient Relations" OR "Patient-Centered Care" OR consent OR chaperone* OR respect* OR dignity OR "care process*" OR explain* OR permission OR modesty OR rapport OR "perceived control" OR "clinician gender" OR "physician gender" OR "examiner gender" OR "sonographer gender" OR preference* OR participation) AND TS=(Trust OR Disclosure OR Anxiety OR Emotions OR "Patient Acceptance of Health Care" OR embarrassment OR shame OR discomfort OR fear OR pain OR distress OR "care avoidance" OR preparedness OR uncertainty OR acceptab*)

Result:1590 Date:2026-9-4

CINAHL

(SU ("pelvic examination*" OR "pelvic exam*" OR "gynecologic examination*" OR "gynaecological examination*" OR "gynecologic exam*" OR "gynaecological exam*" OR "vaginal examination*" OR "vaginal exam*" OR "speculum examination*" OR "speculum exam*" OR "bimanual examination*" OR "intimate examination*" OR "internal pelvic examination*" OR colposcop* OR "transvaginal ultrasound*" OR "transvaginal ultrasonograph*" OR "transvaginal sonograph*") OR AB ("pelvic examination*" OR "pelvic exam*" OR "gynecologic examination*" OR "gynaecological examination*" OR "gynecologic exam*" OR "gynaecological exam*" OR "vaginal examination*" OR "vaginal exam*" OR "speculum examination*" OR "speculum exam*" OR "bimanual examination*" OR "intimate examination*" OR "internal pelvic examination*" OR colposcop* OR "transvaginal ultrasound*" OR "transvaginal ultrasonograph*" OR "transvaginal sonograph*")) AND (SU (communication OR "informed consent" OR consent OR privacy OR "body privacy" OR "bodily privacy" OR chaperone* OR respect* OR dignity OR "continuity of care" OR "care process*" OR "waiting time*" OR explain* OR permission OR modesty OR rapport OR "perceived control" OR "clinician gender" OR "physician gender" OR "examiner gender" OR "sonographer gender" OR preference* OR participation) OR AB (communication OR "informed consent" OR consent OR privacy OR "body privacy" OR "bodily privacy" OR chaperone* OR respect* OR dignity OR "continuity of care" OR "care process*" OR "waiting time*" OR explain* OR permission OR modesty OR rapport OR "perceived control" OR "clinician gender" OR "physician gender" OR "examiner gender" OR "sonographer gender" OR preference* OR participation)) AND (SU (trust OR distrust OR disclosure OR embarrassment OR shame OR discomfort OR anxiety OR fear OR pain OR distress OR "care avoidance" OR reluctance OR preparedness OR uncertainty OR acceptab*) OR AB (trust OR distrust OR disclosure OR embarrassment OR shame OR discomfort OR anxiety OR fear OR pain OR distress OR "care avoidance" OR reluctance OR preparedness OR uncertainty OR acceptab*))

Result:356   Date:2026-9-4

PsycArticles via APA PsycNet

(Any Field: "pelvic examination*" OR Any Field: "pelvic exam*" OR Any Field: "gynecologic examination*" OR Any Field: "gynaecological examination*" OR Any Field: "gynecologic exam*" OR Any Field: "gynaecological exam*" OR Any Field: "vaginal examination*" OR Any Field: "vaginal exam*" OR Any Field: "speculum examination*" OR Any Field: "speculum exam*" OR Any Field: "bimanual examination*" OR Any Field: "intimate examination*" OR Any Field: "internal pelvic examination*" OR Any Field: "gynaecological consultation*" OR Any Field: "gynecologic consultation*" OR Any Field: colposcop* OR Any Field: "transvaginal ultrasound*" OR Any Field: "transvaginal ultrasonograph*" OR Any Field: "transvaginal sonograph*") AND (Any Field: communication OR Any Field: "interpersonal communication" OR Any Field: "doctor-patient communication" OR Any Field: "clinician-patient communication" OR Any Field: "provider-patient communication" OR Any Field: "informed consent" OR Any Field: consent OR Any Field: privacy OR Any Field: "body privacy" OR Any Field: "bodily privacy" OR Any Field: chaperone* OR Any Field: respect* OR Any Field: dignity OR Any Field: "continuity of care" OR Any Field: "care process*" OR Any Field: "care pathway*" OR Any Field: "waiting time*" OR Any Field: accompaniment OR Any Field: support* OR Any Field: trust OR Any Field: distrust OR Any Field: disclosure OR Any Field: "self-disclosure" OR Any Field: embarrassment OR Any Field: shame OR Any Field: discomfort OR Any Field: vulnerab* OR Any Field: anxiety OR Any Field: fear OR Any Field: pain OR Any Field: distress OR Any Field: "care avoidance" OR Any Field: "healthcare avoidance" OR Any Field: "avoidance of care" OR Any Field: reluctance OR Any Field: "delayed attendance" OR Any Field: "delay in care" OR Any Field: "care acceptance" OR Any Field: explain* OR Any Field: permission OR Any Field: modesty OR Any Field: rapport OR Any Field: "perceived control" OR Any Field: "clinician gender" OR Any Field: "physician gender" OR Any Field: "examiner gender" OR Any Field: "sonographer gender" OR Any Field: preference* OR Any Field: participation OR Any Field: preparedness OR Any Field: uncertainty OR Any Field: acceptab*)

Result:5   Date:2026-9-4

CNKI（journal articles）

SU=‘门诊+住院患者+病房+医院门诊+社区卫生服务+妇科门诊+住院+妇科病房+医院+医院妇科+社区妇科服务+妇科门诊服务’AND SU=‘沟通+医患沟通+知情同意+同意+隐私+隐私保护+身体隐私+私密暴露+尊重+尊严+流程+照护过程+信任+不信任+信息披露+症状披露+尴尬+羞耻+不适+脆弱感+焦虑+解释+许可+允许+陪检+陪同+医务人员性别+医生性别+检查者性别+偏好+性别偏好+控制感+感知控制+参与+准备度+准备情况+不确定性+可接受性’AND SU=‘妇科检查+盆腔检查+阴道检查+窥器检查+阴道窥器检查+双合诊+妇科内诊+私密检查+私密照护+妇科诊疗+妇科就诊+妇科门诊+阴道镜检查+阴道镜+经阴道超声+经阴道超声检查+阴道超声+阴道超声检查+经阴道超声成像’

Result:125   Date:2026-9-4

CNKI Dissertations（Grey literature）

SU=‘门诊+住院患者+病房+医院门诊+社区卫生服务+妇科门诊+住院+妇科病房+医院+医院妇科+社区妇科服务+妇科门诊服务’AND SU=‘沟通+医患沟通+知情同意+同意+隐私+隐私保护+身体隐私+私密暴露+尊重+尊严+流程+照护过程+信任+不信任+信息披露+症状披露+尴尬+羞耻+不适+脆弱感+焦虑+解释+许可+允许+陪检+陪同+医务人员性别+医生性别+检查者性别+偏好+性别偏好+控制感+感知控制+参与+准备度+准备情况+不确定性+可接受性’AND SU=‘妇科检查+盆腔检查+阴道检查+窥器检查+阴道窥器检查+双合诊+妇科内诊+私密检查+私密照护+妇科诊疗+妇科就诊+妇科门诊+阴道镜检查+阴道镜+经阴道超声+经阴道超

声检查+阴道超声+阴道超声检查+经阴道超声成像’

Result:5 Date:2026-9-4
