# Supplementary material for: Communication, privacy and interpersonal care in non-obstetric gynaecological examinations: a scoping review
Source: Front Med (Lausanne). 2026 Oct 21;13:1992943. doi: 10.3389/fmed.2026.1992943 (PMC13733400; doi:10.3389/fmed.2026.1992943)
Supplement: Supplementary file 2 [file Datasheet2.pdf]

Supplementary Material 1

PRISMA-ScR Checklist

Section	Item	PRISMA-ScR reporting item	Location in manuscript
TITLE	1	Identify the report as a scoping review.	Article title
ABSTRACT	2	Provide a structured summary covering background, objective, eligibility, sources of evidence, charting/synthesis, results, and conclusions.	Abstract
INTRODUCTION	3	Explain the rationale for the review in the context of existing knowledge and why a scoping approach is appropriate.	Section 1 Introduction; Section 2.1 Review design
INTRODUCTION	4	State the review objective(s) and key concepts clearly.	Section 1 Introduction, final paragraph
METHODS	5	Indicate whether a protocol exists, where it can be accessed, and registration information.	Section 2.2 Protocol registration and reporting. OSF registration DOI: 10.17605/OSF.IO/P8FQW
METHODS	6	Specify eligibility criteria for participants, concept, context, source types, language, and other limits.	Section 2.4 Eligibility criteria and Table 1
METHODS	7	Describe all information sources and the date of the most recent search.	Section 2.3 Search strategy. Final search date: 4 September 2026
METHODS	8	Present the full electronic search strategy for at least one database and make the complete strategies reproducible.	Section 2.3 Search strategy; Supplementary Material 2
METHODS	9	Describe the process for selecting sources of evidence, including reviewer roles and resolution of disagreements.	Section 2.5 Study selection; Figure 1
METHODS	10	Describe the data-charting process, including whether charting was independent/duplicate and how the form was refined.	Section 2.6 Data extraction
METHODS	11	List and define the variables/data items that were charted.	Section 2.6 Data extraction
METHODS	12	If critical appraisal was performed, explain the rationale, methods, and use of appraisal results.	Section 2.8 Methodological quality appraisal; rationale for not conducting formal appraisal
METHODS	13	Describe how the charted data were handled, summarised, and synthesised.	Section 2.7 Data synthesis and evidence mapping
RESULTS	14	Report numbers screened, assessed, excluded (with reasons), and included; preferably use a flow diagram.	Section 3.1 Study selection; Figure 1

Section	Item	PRISMA-ScR reporting item	Location in manuscript
RESULTS	15	Present characteristics of each included source and provide citations.	Section 3.2 Characteristics of included studies; Table 2
RESULTS	16	If critical appraisal was performed, present the appraisal findings.	Not applicable. No formal methodological quality appraisal was conducted (see Section 2.8)
RESULTS	17	For each included source, present the relevant charted findings that contribute to the review questions/objectives.	Table 2; Sections 3.4–3.7
RESULTS	18	Summarise and/or present the synthesis of the charted evidence in relation to the review objectives.	Sections 3.3–3.7; Table 3; Figure 2
DISCUSSION	19	Summarise the main findings, relate them to the review objectives and broader evidence, and discuss their relevance.	Section 4 Discussion
DISCUSSION	20	Discuss limitations of the scoping review process and the evidence base.	Section 6 Limitations
DISCUSSION	21	Provide an overall interpretation and implications, including directions for future research.	Section 5 Clinical implications; Section 7 (Conclusion)
FUNDING	22	Describe funding for the review and, where charted, funding of included sources.	Funding section. Funding of included sources was not charted
